# Effects of Enhanced Resistance and Transcriptome Analysis of Twig Blight Disease by Exogenous Brassinolide in *Myrica rubra*

**DOI:** 10.3390/antiox13010061

**Published:** 2023-12-29

**Authors:** Zheping Yu, Shuwen Zhang, Li Sun, Senmiao Liang, Xiliang Zheng, Haiying Ren, Xingjiang Qi

**Affiliations:** 1 State Key Laboratory for Managing Biotic and Chemical Threats to Quality and Safety of Agro-Products, Institute of Horticulture, Zhejiang Academy of Agricultural Sciences, Hangzhou 310021, China; yuzp@zaas.ac.cn (Z.Y.);; 2 Xianghu Laboratory, Hangzhou 311231, China

**Keywords:** *Myrica rubra*, exogenous brassinolide, twig blight disease, activity of antioxidant enzymes

## Abstract

Twig blight disease is the primary disease that affects the production of *Myrica rubra* in China. It was reported that exogenous brassinolide (BL) can improve disease resistance in plants. Here, we examined the effects of exogenous BL on disease resistance, chlorophyll contents, antioxidant enzyme activity, ROS accumulation, and key gene expression of *M. rubra* to analyze the mechanism of BR-induced resistance of twig blight disease in *M. rubra*. The results demonstrated that 2.0 mg·L^−1^ of BL could significantly lessen the severity of twig blight disease in *M. rubra*. Exogenous BL increased the contents of chlorophyll a, chlorophyll b, carotenoids, and total chlorophyll. Moreover, exogenous BL also significantly enhanced the activity of antioxidant enzymes such as superoxide dismutase (SOD), peroxidase (POD), and catalase (CAT), and decreased malondialdehyde (MDA) content and reactive oxygen species (ROS) accumulation in leaves, such as H_2_O_2_ and O_2_^·−^. Additionally, exogenous BL dramatically up-regulated the expression of pathogenesis-related (PR) genes such as *MrPR1*, *MrPR2*, and *MrPR10*, as well as important genes such as *MrBAK1*, *MrBRI1*, and *MrBZR1* involved in brassinosteroid (BR) signaling pathway. The transcriptome analysis revealed that a total of 730 common differentially expressed genes (DEGs) under BL treatment were found, and these DEGs were primarily enriched in four Kyoto Encyclopedia of Genes and Genomes (KEGG) pathways. Based on these findings, nine important candidate genes related to the resistance of twig blight disease under BL treatment were further identified. In this study, we elucidated the effects of exogenous BL on enhancing the resistance of *M. rubra* to twig blight disease and preliminary analyzed the potential mechanism of resistance induction, which will provide a crucial foundation for the management and prevention of twig blight disease in *M. rubra*.

## 1. Introduction

*Myrica rubra* Sieb. et Zucc. is an economical fruit tree and characteristically distributed in Southern China [1]. The fruit of *M. rubra* is sweet and sour, rich in various nutrients [2], and also possesses the functions of anti-oxidation, anti-aging, and anti-tumor [2,3,4]. So far, twig blight disease is the most severe disease, endangering the sustainable development of the *M. rubra* industry. It was reported that twig blight disease in *M. rubra* is caused by *Pestalotiopsis versicolor* [5], and this disease is characterized by a rapid onset and strong infectiousness. *P. versicolor* is a common endophytic fungus in woody plants and can cause diseases in many commercial crops, such as branch blight of loquats [6], canker and twig dieback of blueberries [7], root and crown rot of strawberries [8], and tea gray blight disease [9].

When encountering pathogens, plants will trigger immune responses, such as pattern-triggered immunity (PTI) and effector-triggered immunity (ETI) [10,11]. During these immune responses, plant hormones can stimulate or induce systemic acquired resistance (SAR) against pathogens, indicating that plant hormones play an important role in immune signal regulation pathways [12,13]. Previous studies showed that plant hormones, such as jasmonic acid (JA), salicylic acid (SA), and brassinosteroids (BRs), not only regulate plant growth and development [14] but also assist plants in coping with biological stresses, such as pests and diseases [15].

BRs are steroidal hormones with physiological activity [16] that play important roles in promoting plant growth and development [17] and enhancing plant responses against abiotic or biotic stresses [18,19]. Exogenous BRs and related compounds can increase antioxidant enzymes activities, such as POD, SOD, and CAT, and reduce MDA content, thus enhancing plant resistance to abiotic stresses, such as drought [20,21], salt [22], and high [23] and low temperature [24]. Furthermore, in plants, BR can alleviate biological stresses caused by pathogens, such as bacteria, fungi, and viruses. For instance, exogenous BR significantly enhanced the resistance of tobacco against tobacco mosaic viruses (TMV), and induced resistance to rice blast and bacterial blight [25]. In plants, researchers have found that exogenous BRs have an impact on metabolites, such as soluble proteins and carbohydrates, when under osmotic stress, and BRs also enhance the accumulation of osmolyte-free proline in radish [26]. Additionally, the application of 24-epibrassinolide (EBL) significantly promoted the antioxidant activity, total anthocyanins, and phenolics contents in strawberries. The results further showed that total anthocyanins were positively correlated with their antioxidant capacity [27]. Resistance induction in plants involves key genes of the BR signaling pathway. Notably, BRI1 and BAK1 are key receptor proteins located on the plasma membrane [28], whereas BZR1 and BES1 are important transcription factors (TFs) involved in the BR signaling pathway. These two TFs regulate the expression of downstream genes through dephosphorylation [29]. It was reported that exogenous BR significantly up-regulated the expression of BR signaling pathway genes in *Malus hupehensis*, such as *MdBRI1*, *MdBAK1*, and *MdBZR1* [17]. In addition, when plants suffer from invasion by pathogens, the expressions of pathogenesis-related proteins (PRs), such as PR1, PR2, and PR10 are strongly induced [30,31], indicating their important roles in the disease resistance of plants [32].

At present, the mechanism of BR-induced twig blight resistance in *M. rubra* remains unclear. This study aimed to investigate the effects of exogenous BR on disease resistance, chlorophyll content, antioxidant enzyme activity, and reactive oxygen species (ROS) accumulation in *M. rubra* leaves after inoculation with the pathogen of twig blight disease. This study also explored the candidate genes associated with resistance induction using RNA-seq analysis. The results of this study will provide a foundation for the prevention and control of twig blight disease in *M. rubra*.

## 2. Materials and Methods

### 2.1. Experimental Materials

Two-year-old healthy ‘Dongkui’ *M. rubra* potted seedlings were used in this study. Each group contained five plants, and all plants were provided with consistent water and fertilizer management. The inducer, brassinolide (BL, the most active BR), was purchased from Shanghai Aladdin Biochemical Technology Co., Ltd, Shanghai, China. A *P. versicolor* strain XJ27 was isolated and provided by the Institute of Horticulture Research, Zhejiang Academy of Agricultural Sciences, Hangzhou, China.

### 2.2. Inoculation

Each plant was sprayed with 100 mL of BL solution (1.0, 2.0, 3.0, and 4.0 mg·L^−1^), and both sides of all leaves were kept moist. Plants of the control group were sprayed with 100 mL of sterilized distilled water. This experiment was carried out in the greenhouse of the Zhejiang Academy of Agricultural Sciences (120°11′ E, 30°18′ N) and involved three spray treatments on 5 April, 12 April, and 19 April 2022.

Following the method of [5], leaves were inoculated with strain XJ27 on 3 May 2022 (14 days after the third spray). Mature leaves (20 pieces per plant) were selected, and the mycelial blocks were placed on the surface of punctured leaves for infection. Subsequently, diseased areas of leaves were measured using ImageJ software, and the mycelium growth was observed using ultra-depth field microscopy (VHX-5000, Keyence(China) Co., Ltd., Shanghai, China).

### 2.3. Determination of Chlorophyll Content

The ethanol extraction method was used to determine the chlorophyll content of leaves [33]. Leaves were collected, fully cut, and mixed (0.2 g) with 10 mL of 95% ethanol. These soaked leaves were kept in the dark for 48 h, with multiple shakings. The optical density (OD) was then determined at 470 nm, 645 nm, and 663 nm using a visible light spectrophotometer, and the content of chlorophyll a (Chl a), chlorophyll b (Chl b), total chlorophyll (Chl), and carotenoid (Caro) was calculated using the formula:

ChI a = (12.7 *A*_663nm_ − 2.69 *A*_645nm_) × V/(W × 1000)

ChI b = (22.9 *A*_645nm_ − 4.68 *A*_645nm_) × V/(W × 1000)

Caro = (1000 *A*_470nm_ − 3.27 ChI a − 104 ChI b) × V/(229 × W × 1000)

ChI = ChI a + ChI b

In the formulas above, *A*_663nm_, *A*_645nm_, and *A*_470nm_ represent OD values obtained at OD of 663 nm, 645 nm, and 470 nm, respectively, whereas V and W represent extraction liquid volume (mL) and leaf mass (g), respectively.

### 2.4. Determination of Antioxidant Enzyme Activity and ROS Accumulation

Leaves (0.5 g) were weighed, and mixed with 0.05 M phosphate-buffered saline (PBS), and the mixture was centrifuged at 10,000 rpm for 10 min at 4 °C. The supernatant was used to measure the activity of different antioxidant enzymes. SOD activity was determined using the nitrogen blue tetrazole (NBT) method [34]. POD activity was measured using visible spectrophotometry [35], whereas CAT activity was determined using the ultraviolet absorption method [36]. The MDA content was estimated using total bile acids (TBA) colorimetry [37]. The determination of ROS accumulation used the method by [38]. In this method, H_2_O_2_ and O_2_^·−^ contents were determined using visible spectrophotometry. All kits were purchased from Suzhou Comin Biotechnology Co., Ltd., Suzhou, China.

### 2.5. qRT-PCR Analysis

Collected leaves were quickly frozen in liquid nitrogen and stored at −80 °C. RNA was extracted using a rapid RNA extraction kit (DP452), and the RNA was reverse-transcribed using a reverse transcriptase kit (NG212). A qRT-PCR test was performed using FastFire qPCR PreMix (SYBR Green). The kits and enzymes were purchased from Tiangen Biotech (Beijing) Co., Ltd., Beijing, China. The reaction of qRT-PCR reaction (10 μL) involved a pre-denaturation step at 95 °C for 1 min, reaction steps at 95 °C for 5 s and 58 °C for 10 s, and a prolongation step at 72 °C for 15 s, with a total of 35 cycles. The *MrActin* gene was used as the internal control. The qRT-PCR reaction was conducted three times for each sample, and the relative expression of relevant genes was calculated using the 2^−ΔΔCt^ method [39]. Vector NTI version 11.5.3 was used to design the primers (Table S1).

### 2.6. RNA-seq Analysis

Transcriptomics was performed by Biomarker Technologies (BMK). The BMK Cloud platform (www.biocloud.net, accessed on 23 July 2022) was used to perform the analysis of differentially expressed genes (DEGs), principal component analysis (PCA), Venn diagram, and correlation analysis. Gene Ontology (GO) enrichment analysis of DEGs was implemented by the GOseq R packages based on Wallenius non-central hyper-geometric distribution [40]. The analysis of Kyoto Encyclopedia of Genes and Genomes (KEGG) pathway enrichment was performed by KOBAS version 3.0 [41].

### 2.7. Data Analysis

All the data was statistically analyzed using Microsoft Excel 2013, and SPSS version 18.0 software was used to perform a one-way analysis of variance (ANOVA).

## 3. Results

### 3.1. Exogenous BL Significantly Reduces the Level of Twig Blight Disease in M. rubra Leaves

**Figure 1:**
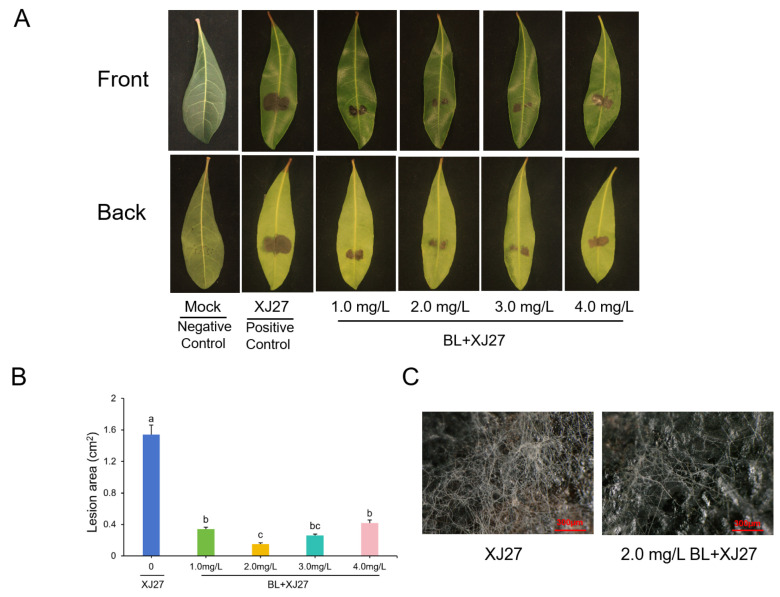
Phenotypes of *M. rubra* leaves under BL treatment after inoculation with pathogen of twig blight disease. (**A**) Leaf incidence under treatment of different BL concentrations. (**B**) Lesion area of leaves caused by XJ27. (**C**) The mycelial growth of positive control and BL treatment group. Different lowercase letters indicate significant differences among different treatments at 0.05 level. Mock represents the negative control; XJ27 represents the positive control, inoculated with the pathogen of twig blight disease (strain XJ27); and BL+XJ27 represents BL treatment before inoculation.

In this study, we first sprayed BL on leaves and then infected them with the strain XJ27. It was found that the lesion area of leaves treated with BR after inoculation was significantly smaller than that of the positive control (XJ27 group) (Figure 1A). As the BL concentration increased, the lesion area of leaves first decreased and then subsequently increased, with the smallest lesion area observed at a BR concentration of 2.0 mg·L^−1^ (Figure 1B). At this BL concentration, the condition at the backside of the leaves was consistent with that at the frontside, indicating 2.0 mg·L^−1^ as the optimal concentration for BL treatment (Figure 1A). The mycelial growth on the surfaces of leaves was observed using an ultra-depth field microscope, and its growth on the surface of leaves treated with 2.0 mg·L^−1^ of BL was significantly weaker than that of leaves of the positive group (Figure 1C). These results suggested that pretreatment with BL could significantly reduce the level of twig blight disease in *M. rubra* leaves. This also indicated that BL might improve the resistance of *M. rubra* to twig blight disease.

### 3.2. Exogenous BL Treatment Increases Chlorophyll Content in M. rubra Leaves

**Figure 2:**
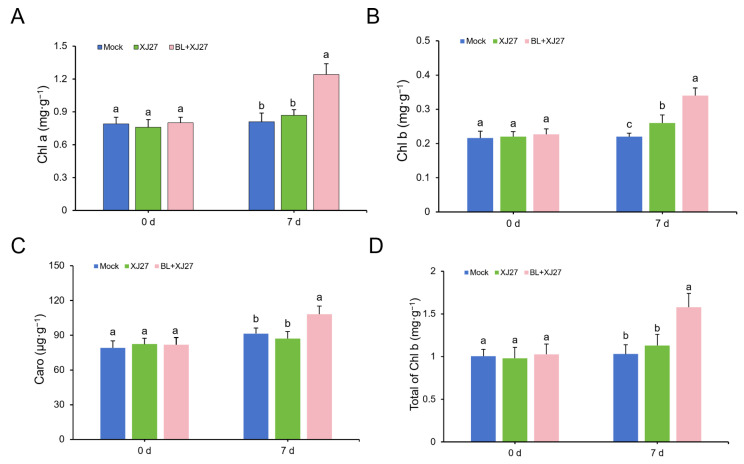
Chlorophyll contents of *M. rubra* leaves under BL treatment after inoculation with pathogen of twig blight disease. (**A**) Content of Chl a. (**B**) Content of Chl b. (**C**) Content of Caro. (**D**) Total Chl content. Different lowercase letters indicate significant differences among different treatments at 0.05 level. Mock represents the negative control; XJ27 represents the positive control, inoculated with the pathogen of twig blight disease (strain XJ27); BL+XJ27 represents BL treatment before inoculation; and 7 d represents 7 days after inoculation.

Since 2.0 mg·L^−1^ of BL resulted in the best resistance induction effect in *M. rubra*, further experiments were conducted using this BL concentration. We determined the chlorophyll content of infected leaves and found that 7 d after inoculation with the pathogen of twig blight disease, the Chl a content under BR treatment was increased by 42.5% when compared with that of the positive control (XJ27) (Figure 2A). Furthermore, the content of Chl b and Caro was 0.34 mg·g^−1^ and 108.27 μg·g^−1^, respectively, which were 30.77% and 24.24% higher than those of the positive control, respectively (Figure 2B,C). Consistent results were obtained for the total chlorophyll content (Figure 2D). Hence, exogenous BL treatment increased the chlorophyll content of leaves inoculated with the pathogen of twig blight disease in *M. rubra*.

### 3.3. Exogenous BL Increases Antioxidant Enzyme Activity and ROS Accumulation in M. rubra

**Figure 3:**
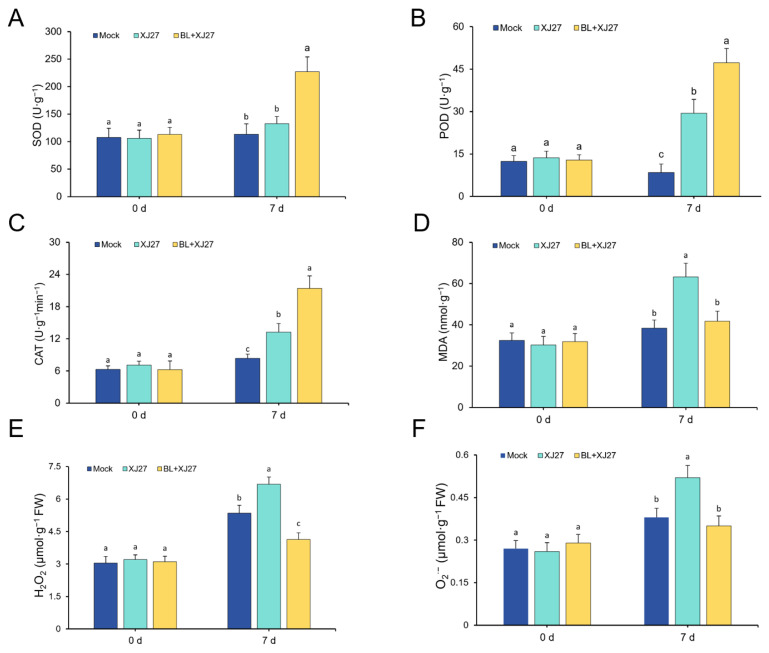
Effects of exogenous BL on antioxidant enzyme activity and ROS accumulation in *M. rubra*. (**A**) SOD activity. (**B**) POD activity. (**C**) CAT activity. (**D**) MDA content. (**E**) H_2_O_2_ content. (**F**) O_2_^·−^ content. Different lowercase letters indicate significant differences among different treatments at 0.05 level. Mock represents the negative control; XJ27 represents the positive control, inoculated with the pathogen of twig blight disease (strain XJ27); BL+XJ27 represents BR treatment before inoculation; and 7 d represents 7 days after inoculation.

When treated with BL, the activity of SOD significantly increased, reaching 227.06 U·g^−1^ on day 7, and was 71.07% higher than that of the positive control (XJ27) (Figure 3A). Compared with the positive control, POD and CAT activities also increased significantly by 60.69% and 61.56%, respectively on day 7 (Figure 3B,C). In contrast, the MDA content under BL treatment was notably decreased and was 51.29% lower than that of the positive control 7 d after inoculation with the pathogen of twig blight disease (Figure 3D).

The effect of exogenous BL on ROS accumulation in leaves was further analyzed. The H_2_O_2_ content of the positive control on day 7 was significantly increased, reaching 6.68 μmol·g^−1^·FW, which was 24.86% higher than that of the mock. The group under BL treatment demonstrated a remarkably decreased H_2_O_2_ content (38.17% lower than that of the positive control) (Figure 3E). A similar trend was observed for the O_2_^·−^ content in BL-treated and non-treated leaves. The O_2_^·−^ content under exogenous BL treatment on day 7, had a 32.70% decrease compared with the positive control (Figure 3F).

These results indicated that exogenous BL treatment could significantly enhance the activities of SOD, POD, and CAT, and reduce MDA content in leaves. Additionally, BL treatment notably reduced the levels of H_2_O_2_ and O_2_^·−^ in *M. rubra* leaves, which means that exogenous BL can decrease the accumulation of ROS to better resist the oxidative damage caused by twig blight disease.

### 3.4. Effects of Exogenous BR on Expression of PR Genes and BR Signaling-Related Genes

**Figure 4:**
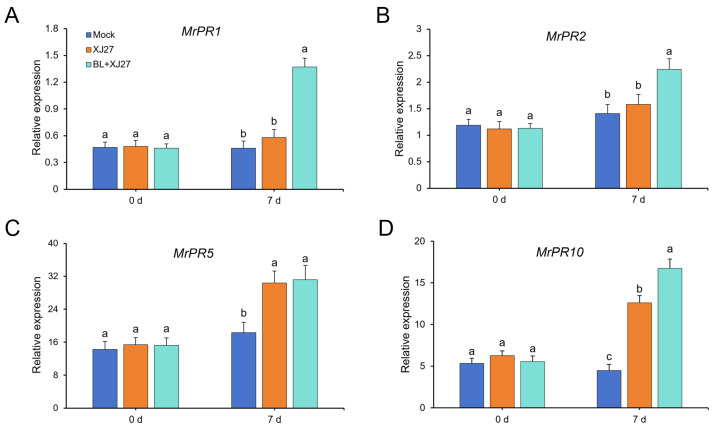
Effects of exogenous BL on the expression of pathogenesis-related gene (PR) genes in *M. rubra* after inoculation. (**A**) *MrPR1*. (**B**) *MrPR2*. (**C**) *MrPR5*. (**D**) *MrPR10*. Different lowercase letters indicate significant differences among different treatments at 0.05 level. Mock represents the negative control; XJ27 represents the positive control, inoculated with the pathogen of twig blight disease (strain XJ27); BL+XJ27 represents BL treatment before inoculation; and 7 d represents 7 days after inoculation.

qRT-PCR was performed to evaluate the expression of pathogenesis-related gene (PR) genes (*MrPR1*, *MrPR2*, *MrPR5*, and *MrPR10*) in *M. rubra* after exogenous BR treatment (Figure 4). The expression of *MrPR1* under BL treatment (BL+XJ27) on day 7 was significantly increased and 2.36 times higher than that of the positive control (XJ27) (Figure 4A). Similarly, the expressions of *MrPR2* and *MrPR10* under BL treatment were increased by 1.42 and 1.33 times, when compared with those of the positive control (Figure 4B,D). Although the expression of *MrPR5* in positive control and under BL treatment was significantly higher (1.55 and 1.65 times, respectively) than that of the mock, there was no obvious difference between these two treatments (Figure 4C). Hence, exogenous BL significantly upregulated the expressions of *MrPR1*, *MrPR2,* and *MrPR10* in *M. rubra* after inoculation with the pathogen of twig blight disease.

**Figure 5:**
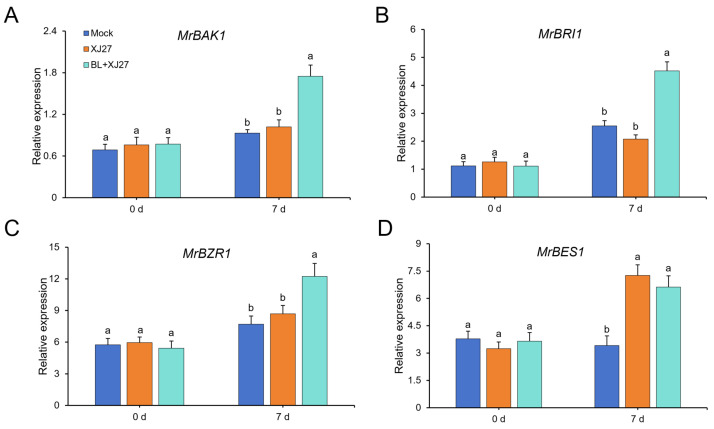
Effects of exogenous BL on the expression of key genes involved in BR-signal pathway in *M. rubra* after inoculation. (**A**) *MrBAK1*. (**B**) *MrBRI1*. (**C**) *MrBZR1*. (**D**) *MrBES1*. Different lowercase letters indicate significant differences among different treatments at 0.05 level. Mock represents the negative control; XJ27 represents the positive control, inoculated with the pathogen of twig blight disease (strain XJ27); BL+XJ27 represents BL treatment before inoculation; and 7 d represents 7 days after inoculation.

The influence of exogenous BL treatment on the expression of key genes (*MrBAK1*, *MrBRI1*, *MrBZR1*, and *MrBES1*) involved in the BR signaling pathway was further studied (Figure 5). The expression of *MrBAK1* under BL treatment on day 7 was significantly upregulated and 1.72 times higher than that in the positive control, but no obvious difference was observed for the expression of *MrBAK1* between the positive control and mock (Figure 5A). Similar results were obtained for *MrBRI1* and *MrBZR1*. The expression of *MrBRI1* and *MrBZR1* under BL treatment on day 7 was significantly upregulated and increased by 1.17 and 0.41 times compared with that of the positive control, respectively (Figure 5B,C). Furthermore, the expression of *MrBES1* in the positive control was significantly higher than that of mock, and it was slightly lower under BR treatment than that of the positive control, with no obvious difference (Figure 5D).

### 3.5. RNA-seq Analysis of M. rubra Leaves under Exogenous BR Treatment

#### 3.5.1. Transcriptome Data Quality Analysis

Nine samples were used for the transcriptome sequence. High-throughput sequencing of the samples was performed using the Illumina NovaSeq 6000 platform. The data were filtered to produce clean data consisting of 185.26 M Reads, totaling 55.43 Gb. The ratio of Q30 bases was higher than 92.09%, and the ratio of GC bases was up to 46.26–46.90% of the total base number (Table S2), which suggested that the sequencing quality of the samples is relatively high and could meet the requirement of bioinformatics.

#### 3.5.2. Analysis of DEGs

**Figure 6:**
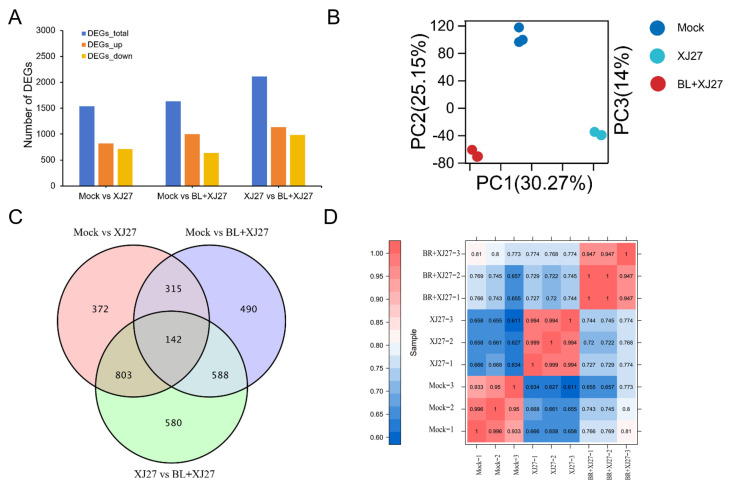
DEGs analysis of *M. rubra* leaves inoculated with the pathogen of twig blight disease under exogenous BL. (**A**) Numbers of DEGs. (**B**) PCA analysis. (**C**) Venn Diagram of DEGs. (**D**) correlation analysis of transcriptome samples. Mock represents the negative control; XJ27 represents the positive control, inoculated with the pathogen of twig blight disease (strain XJ27); and BL+XJ27 represents BL treatment before inoculation.

Gene expressions of samples were assessed using the FPKM method, and differential expression analysis among the different treatment groups was conducted by DESeq. An FDR of <0.01 and a Fold change (FC) of ≥2 or ≤−2 were taken as the screening criteria for DEGs. Between the mock and positive control, a total of 1535 DEGs, including 822 up-regulated and 713 down-regulated genes, were screened out. Compared to the mock, a total of 1632 DEGs, including 998 up-regulated and 634 down-regulated, were screened out under BR treatment. There were 2113 DEGs in total, consisting of 981 down-regulated and 1132 up-regulated genes, which were screened out between positive control and BR treatment (Figure 6A).

According to the results of PCA, three replicates for the same treatment were all able to cluster together, demonstrating the good consistency of transcriptome samples. The contribution of three principal components, PC1, PC2, and PC3, was 30.27%, 25.15%, and 14.0%, respectively (Figure 6B). A Venn diagram revealed that 142 DEGs were spread throughout the different treatments (Figure 6C). The correlation coefficient *r*^2^ among replicates in the same group was greater than 0.933, indicating a strong correlation between replicates (Figure 6D). There was a relatively high gene difference between positive control and BL treatment, as indicated by the comparatively small *r*^2^ between these two groups, which varied from 0.720 to 0.774.

#### 3.5.3. GO Enrichment Analysis

**Figure 7:**
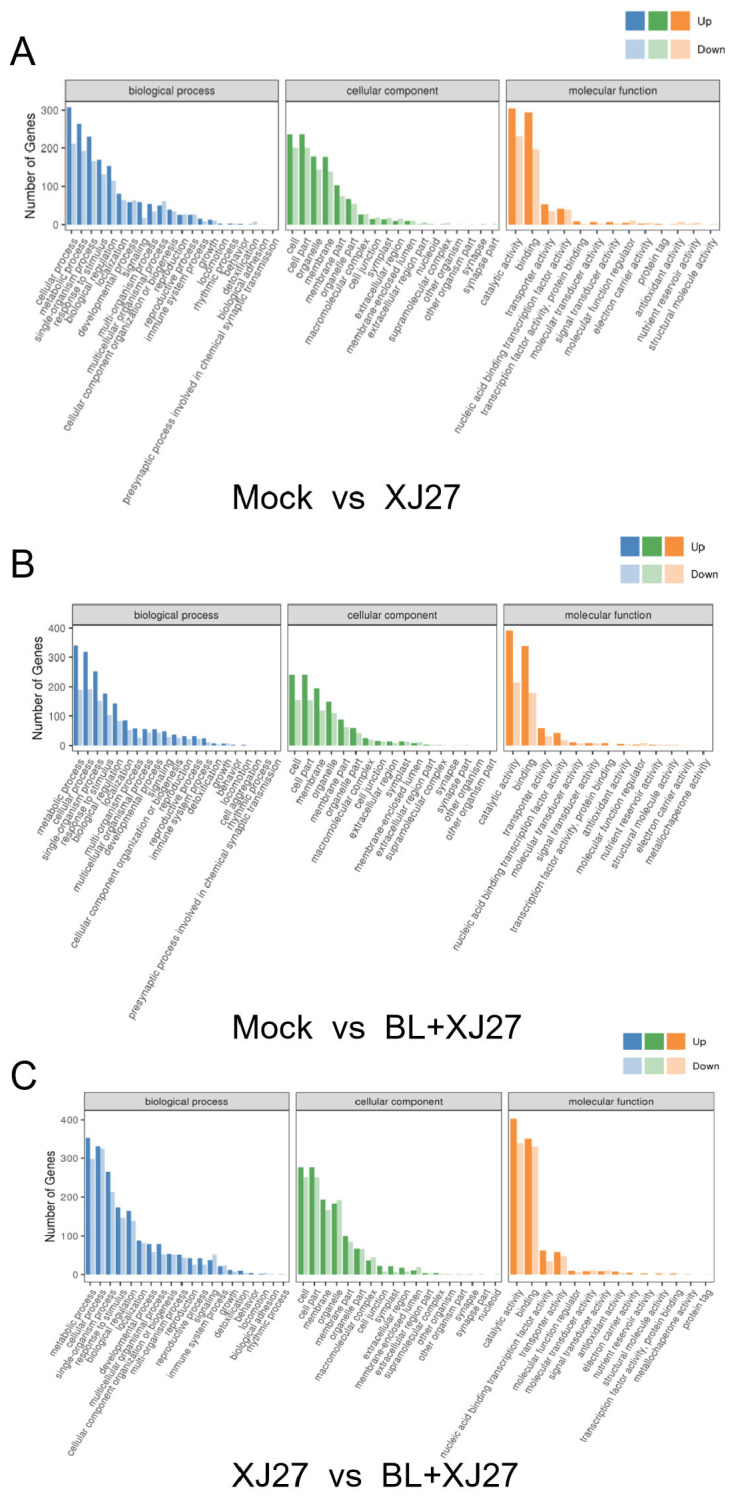
GO enrichment analysis of DEGs of *M. rubra* leaves inoculated with the pathogen of twig blight disease under BL treatment. (**A**) CK vs. XJ27. (**B**) CK vs. BL+XJ27. (**C**) XJ27 vs. BL+XJ27. Mock represents the negative control; XJ27 represents the positive control, inoculated with the pathogen of twig blight disease (strain XJ27); and BL+XJ27 represents BL treatment before inoculation.

Following various treatments, the gene ontology (GO) enrichment analysis of DEGs was conducted, and the quantity of DEGs in each of the three GO categories was annotated to various items (Figure 7). The results demonstrated that DEGs between the positive control and BR treatment were mainly concentrated in biological processes, including cellular processes, metabolic processes, and single-organism processes. In terms of cellular components, DEGs were primarily enriched in components such as cells, cell parts, and membranes. Molecular functions, such as catalytic activity and binding, had relatively more DEGs (Figure 7C). GO enrichment analysis of DEGs for mock vs. XJ27 and XJ27 vs. BR+XJ27 are shown in Figure 7A and Figure 7B, respectively.

#### 3.5.4. KEGG Enrichment Analysis

**Figure 8:**
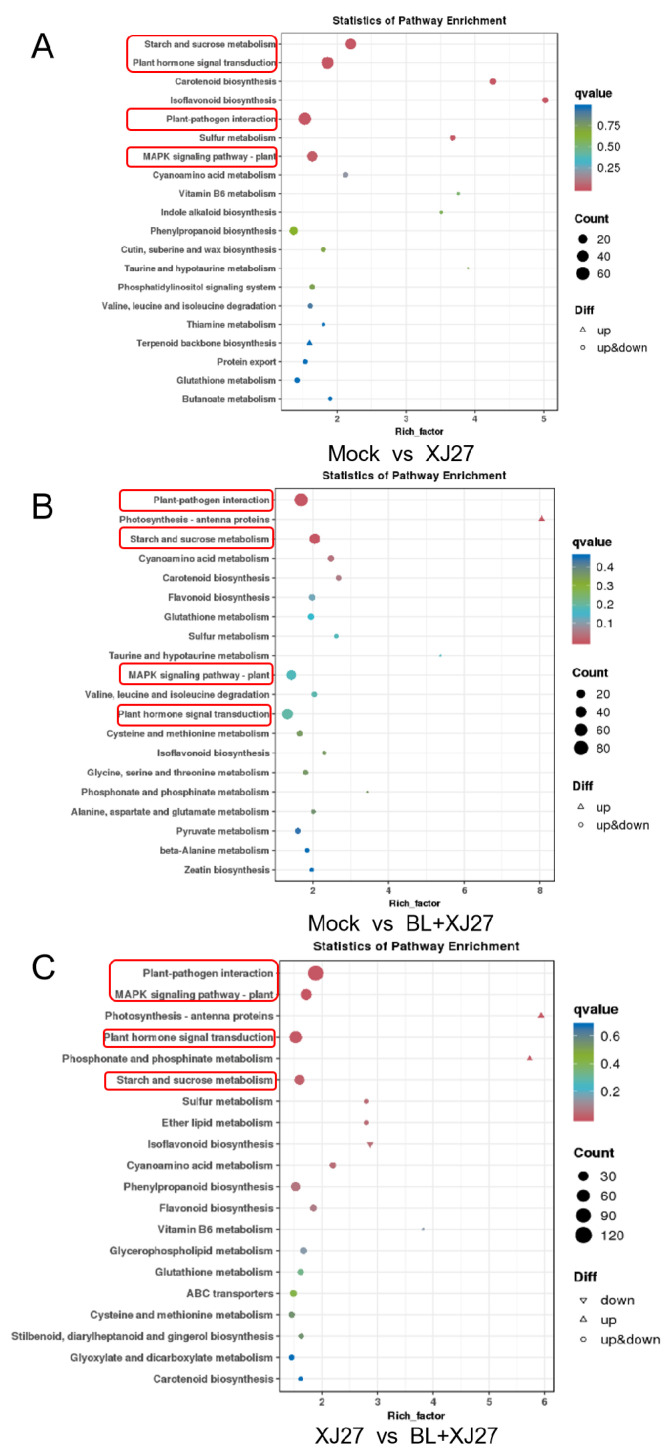
KEGG enrichment analysis of DEGs of *M. rubra* leaves inoculated with the pathogen of twig blight disease under BL treatment. (**A**) CK vs. XJ27. (**B**) CK vs. BL+XJ27. (**C**) XJ27 vs. BL+XJ27. Mock represents the negative control; XJ27 represents the positive control, inoculated with the pathogen of twig blight disease (strain XJ27); and BL+XJ27 represents BL treatment before inoculation; The red boxes represents the four major KEGG pathways.

KEGG enrichment analysis of metabolic pathways was performed for DEGs under different treatments. A total of 738 DEGs between XJ27 and BL+XJ27 treatments were annotated to 123 KEGG metabolic pathways. The plant-pathogen interaction pathway, which enriched 120 DEGs in total (65 up-regulated and 55 down-regulated), was the most abundant among these pathways. Following the plant-pathogen interaction, pathways of plant hormone signal transduction, plant MAPK signaling, and starch and sucrose metabolism had relatively more DEGs, reaching 72, 58, and 46, respectively (Figure 8C). The KEGG enrichment results for mock vs. XJ27 and mock vs. BL+XJ27 were consistent with that of XJ27 vs. BL+XJ27, and the DEGs were mainly concentrated in the above four pathways, with most concentrated in the plant-pathogen interaction (Figure 8A,B).

#### 3.5.5. Identification of Candidate Genes Responding to BR in *M. rubra*

**Figure 9:**
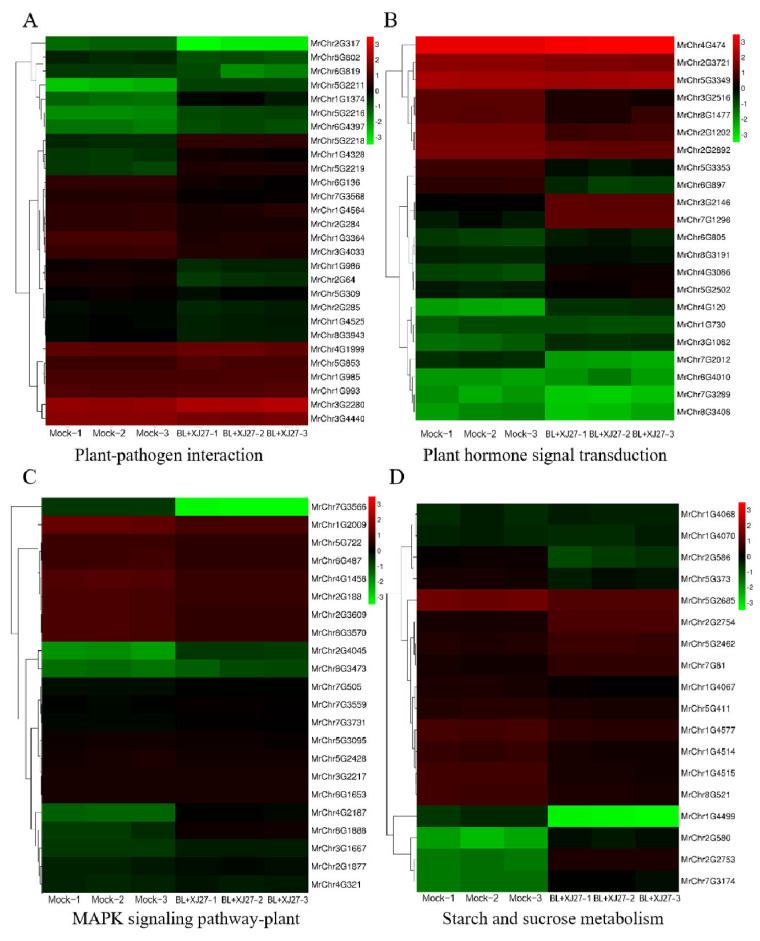
Expression patterns of BL-responsive DEGs of *M. rubra* in major KEGG pathways. (**A**) Expression of DEGs in plant-pathogen interaction. (**B**) Expression of DEGs in plant hormone signal transduction. (**C**) Expression of DEGs in plant MAPK signaling pathways. (**D**) Expression of DEGs in starch and sucrose metabolism.

Based on the above four major KEGG metabolic pathways, key candidate genes responding to BR in *M*. *rubra* were further explored. Previously, 730 DEGs for XJ27 vs. BL+XJ27 had been identified (Figure 6C). On that basis, 29, 22, 22, and 18 DEGs were screened from the pathways of plant-pathogen interaction, plant hormone signal transduction, plant MAPK signaling, and starch and sucrose metabolism, respectively. The corresponding gene expression is displayed in Figure 9. Taking an FC of ≥3 or ≤−3 as screening criteria, two genes (*MrChr2G317*, *MrChr5G2211*) were identified among 29 DEGs in the plant-pathogen interaction pathway. In other major pathways, one (*MrChr4G120*), one (*MrChr7G3566*), and five (*MrChr1G4499*, *MrChr2G2753*, *MrChr2G2754*, *MrChr2G580*, and *MrChr7G3174*) genes were identified, respectively. Next, we will further study the important roles of the above key candidate genes in the regulation of exogenous BR on the resistance of *M. rubra* against twig blight disease.

## 4. Discussion

As a significant plant hormone, BR can induce and strengthen plant resistance to disease, and promote plant growth and development [17,42]. In the current study, it was discovered that prior application of exogenous BL to *M. rubra* leaves inoculated with blight bacteria resulted in a significant reduction in the degree of twig blight disease and a partial decrease in the number of mycelia. Interestingly, it was discovered that BL-induced twig blight resistance of *M. rubra* had a concentration effect based on the concentration gradient test findings of exogenous BL. Specifically, *M. rubra* treated with low concentrations of BL (≤3.0 mg/L) could improve the resistance to twig blight disease, while treated with high concentrations of BL would be somewhat less resistant. This broadly aligns with the findings of the investigation into how exogenous BL regulated tomato resistance to the southern root-knot nematode [43]. Currently, the most severe disease affecting the production of *M. rubra* is twig blight disease. This study will provide a crucial foundation for the comprehensive prevention and control of twig blight disease by the application of BL.

Previous studies have demonstrated that BRs participated in many physiological processes in plants, such as promoting the accumulation of chlorophyll. It was found that when treated with 0.1 μM of EBR, the Chl (a+b) content in leaves of maize seedlings was increased by 7.4% [44]. Further, both the photosynthetic rate and the contents of Chl a and Chl b in tomatoes were significantly increased when treated with the combination of SA and EBR [45]. The results of the present study were consistent with those of earlier investigations. The levels of Chl a, Chl b, Caro, and total chlorophyll in *M. rubra* leaves dramatically increased on day 7 after inoculation under BL treatment, indicating that exogenous BL can promote the accumulation of chlorophyll in plants and assist in the improvement of plant resistance by indirectly enhancing plant photosynthesis.

When plants are subjected to various stresses, the oxidative damage caused by stress can be alleviated by altering the activity of the antioxidant system in plants [46]. Research has indicated that during the initial phases of stress exposure, the activity of the antioxidant system in plants was markedly increased [47,48,49]. After applying exogenous BRs to *Arabidopsis* seedlings, it was discovered that the improvement in the activity of four antioxidant enzymes (SOD, POD, CAT, and APX) could strengthen the plant’s resistance to CMV [50]. The present study determined the amount of MDA in leaves as well as the activity of SOD, POD, and CAT enzymes. It was found that the activities of three antioxidant enzymes were significantly increased when treated with BL 7 d after inoculation with the pathogen of twig blight disease, all of which were increased by more than 60% as compared to the control group, while MDA content was significantly decreased. These findings suggested that exogenous BL treatment could quickly activate the activity of the antioxidant enzyme system in *M. rubra* to better cope with disease stress. It has been found that when suffering from stress, plants quickly produce a large amount of ROS, including major components such as H_2_O_2_ and O_2_^·−^ [51], but they also tend to reduce the oxidative damage caused by ROS by enhancing the activity of antioxidant system [52,53]. In the present study, 7 d after inoculation under exogenous BL treatment, significant decreases in H_2_O_2_ and O_2_^·−^ content were observed, indicating that antioxidant enzymes played an important role in the clearance of intracellular ROS.

When plants are exposed to pathogen stress, PR genes such as *PR1* and *PR2* [30,31] can be strongly activated. These genes are an essential component of the plant defense system, and crucial for the downstream plant disease resistance response. The expression of four PR genes in *M. rubra* (*MrPR1*, *MrPR2*, *MrPR5*, and *MrPR10*) was examined in this study. Exogenous BL treatment resulted in a considerable up-regulation of *MrPR1*, *MrPR2*, and *MrPR10*, but did not change the expression levels of *MrPR5* compared to the positive control. These results demonstrated that during the infection process of the twig blight disease pathogen, exogenous BL significantly induced the expression of PR genes, which indirectly indicated that exogenous BL enhanced the resistance of plants to pathogens. To further determine the potential involvement of key genes in the reaction process of exogenous BL enhancing *M. rubra* resistance, four key genes in the BR signaling pathway were selected: *MrBAK1*, *MrBRI1*, *MrBZR1*, and *MrBES1*. The results of qRT-PCR demonstrated that *MrBAK1*, *MrBRI1,* and *MrBZR1* were all significantly up-regulated under exogenous BL treatment, indicating the close relationship between BR signaling genes and exogenous BL-induced resistance.

Transcriptome analysis employing high-throughput sequencing technologies can assist researchers in the identification of important genes related to plant disease resistance through functional analysis of DEGs. The field of plant disease resistance has made extensive use of transcriptome sequencing [54,55,56]. In this study, based on the confirmation that exogenous BL improves the resistance of *M. rubra* to twig blight disease, RNA-seq was used to mine DEGs closely associated with BL treatment. A total of 730 DEGs for XJ27 vs. BL+XJ27 were discovered. Further, according to GO functional enrichment analysis and KEGG metabolic pathway enrichment analysis, these DEGs were primarily enriched in the plant-pathogen interaction, plant hormone signal transduction, plant MAPK signaling, and starch and sucrose metabolism pathways. Nine important candidate genes (including *MrChr2G317*, *MrChr5G2211*, etc.), were further discovered when combined with gene expressions. Further investigation into the modulation of resistance in *M. rubra* twig blight by exogenous BL will be greatly aided by these potential genes.

Previous studies have proposed several methods for the control of twig blight disease in *M. rubra*. Ahmed et al. found that rhizospheric *Bacillus* has potential inhibition effects against *P. versicolor* XJ27, especially for two strains of *B. siamensis S3* and *B. tequilensis S5*, which showed the strongest effects [57]. Moreover, researchers also proposed a novel method for the control of *P. versicolor* by the use of native *Enterobacter* sp. strain containing biologically synthesized zirconium oxide nanoparticles (ZrONPs) [58]. In this study, we found that exogenous BL possessed significant resistance-enhancing effects against *P. versicolor* in *M. rubra*. Compared with the previous methods, this BL method has certain advantages. The major benefit is that BL can induce *M. rubra* to develop immune resistance by promoting the activity of antioxidant enzymes and up-regulating the expression of PR genes; this kind of induced resistance is durable and broad-spectrum. Thus, the function of BL is similar to the effects of ‘vaccines’. Regarding the methods of rhizospheric *Bacillus* and ZrONPs, although both of them displayed strong inhibitions of *P. versicolor*, their mechanisms were mainly through the direct inhibitory effects on the pathogen of *P. versicolor*, such as the hydrolytic enzymes and lipopeptides produced by *Bacillus* [57], the extracellular leakage of DNA, and proteins caused by ZrONPs [58]. However, the robustness of the BL method on the resistance-induced effects on *P. versicolor* still needs to be investigated in other main varieties of *M. rubra* and more field experiments in the future.

## 5. Conclusions

**Figure 10:**
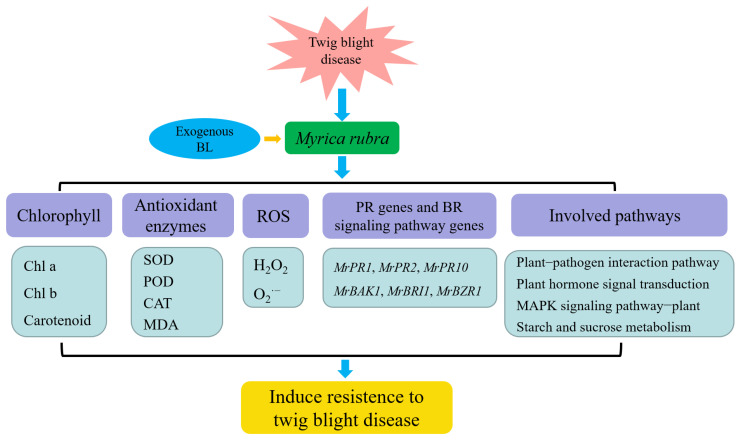
Hypothetical model of exogenous BL to induce the resistance to twig blight disease in *M. rubra*.

Exogenous BL dramatically lessened the severity and induced the resistance of twig blight disease in *M. rubra*, increased chlorophyll contents in leaves, significantly enhanced the activity of antioxidant enzymes, and decreased ROS accumulation. The PR genes *MrPR1*, *MrPR2*, and *MrPR10*, as well as BR signaling pathway genes *MrBAK1*, *MrBRI1,* and *MrBZR1*, were all up-regulated by exogenous BL. The KEGG enrichment analysis showed that DEGs were primarily enriched in pathways such as plant-pathogen interactions and plant hormone signal transduction, and RNA-seq analysis further revealed nine candidate genes associated with resistance to twig blight disease. A hypothetical model of exogenous BL-mediated resistance of twig blight disease in *M. rubra* is displayed in Figure 10. In this study, we first reported the resistance-induced effect of BL on the twig blight disease in *M. rubra*, and the detailed regulation mechanism and the identification of key metabolites responsible for the antioxidant activity of BL need to be further studied in future work.

## Data Availability

All of the data is contained within the article.

## Supplementary Materials

The following supporting information can be downloaded at: https://www.mdpi.com/article/10.3390/antiox13010061/s1, Table S1: Primer sequences for qRT-PCR; Table S2: Sequence reads of the transcriptome and their alignment with the reference genome.
