# Supplementary material for: Effects of Enhanced Resistance and Transcriptome Analysis of Twig Blight Disease by Exogenous Brassinolide in Myrica rubra
Source: Antioxidants (Basel). 2023 Dec 29;13(1):61. doi: 10.3390/antiox13010061 (PMC10812535; doi:10.3390/antiox13010061)
Supplement: Supplementary file 1 [file antioxidants-13-00061-s001.zip › antioxidants-2782809-supplementary.pdf]

Table S1. Primer sequences for qRT-PCR.

Gene	Forward primer	Reverse primer
MrActin	AATGGAACTGGAATGGTCAAGGC	TGCCAGATCTTCTCCATGTCATCCCA
MrPR1	AGAGGTGGGTGTTGGACCTA	GCTGCTCCATGCGATGTTTT
MrPR2	GCTCAGAGGGAGCTGCAAA	CACTCCCCACCAATACACGA
MrPR5	GGAGTTCTCTCCGGAAACGG	CCACAATCACCGGTAAGGCA
MrPR10	CGGATGGTTCACTCCTACGG	ATTTGACCGGGATCGCAGAA
MrBAK1	ATGGCACCAGAATCGCTGTT	ATGCACCCTCAACGCAGTAT
MrBRI1	GGTCAACCTCTGCATACCGT	CCAGCAATCTCCTCGACTCC
MrBZR1	AGTGGGCAAGTTCCATGCTT	GAGCTACACAAGCCATTGCG
MrBES1	TGGCTCCTGAGTTGTTCTACA	AGTTTGGCTTGAATGGTCTGC

Table S2. Sequence reads of the transcriptome and their alignment with the reference genome.

Sample	Obtained Reads (M)	Obtained Bases(Gb)	Q20(%)	Q30(%)	GC(%)
Mock-1	21.31	6.35	98.38	95.43	46.63
Mock-2	20.45	6.12	98.19	94.85	46.46
Mock-3	21.41	6.41	97.45	93.06	46.75
XJ27-1	19.89	5.95	97.61	93.29	46.26
XJ27-2	19.53	5.85	97.72	93.56	46.32
XJ27-3	21.93	6.57	98.05	94.31	46.26
BL+XJ27-1	20.76	6.21	98.22	94.94	46.49
BL+XJ27-2	20.57	6.16	98.29	95.08	46.47
BL+XJ27-3	19.41	5.81	97.04	92.09	46.9
